# Analysis of kinetic parameters of sexed Holstein-Friesian bull spermatozoa using Percoll density gradient centrifugation with computer-assisted sperm analysis

**DOI:** 10.14202/vetworld.2025.287-295

**Published:** 2025-02-13

**Authors:** Putri Utami, Aulia Puspita Anugra Yekti, Chairun Nisa Aperi Simbolon, Habib Asshidiq Syah, Anny Amaliya, Tri Agus Siswoyo, Nurul Isnaini, Trinil Susilawati

**Affiliations:** 1 Department of Animal Reproduction and Breeding, Faculty of Animal Science, Universitas Brawijaya, Malang, Indonesia; 2 Singosari Artificial Insemination Center (SAIC), Malang, Indonesia; 3 Graduate Program of Biotechnology, the Center of Excellence on Crop Industrial Biotechnology (PUI-PT BioTIn), Universitas Jember, Jember, Indonesia

**Keywords:** Artificial insemination, computer-assisted sperm analysis, Holstein-Friesian, Percoll density gradient centrifugation, sperm motility

## Abstract

**Background and Aim:**

Artificial insemination (AI) is a key biotechnology for improving dairy cattle populations, offering genetic enhancement and increased milk production. The advent of sexed semen allows for the preferential selection of female offspring which is beneficial for dairy operations. This study aimed to evaluate and optimize a spermatozoa sexing method using Percoll density gradient centrifugation (PDGC) and analyze kinetic parameters of the separated spermatozoa using computer-assisted sperm analysis.

**Materials and Methods:**

The study was conducted on two Holstein-Friesian bulls at the Singosari Artificial Insemination Center and Universitas Brawijaya, Indonesia. Semen samples underwent PDGC sexing at two density gradients, (T1) 20%-65% and (T2) 20%-60%. Kinetic parameters, including motility, velocity, and movement patterns, were assessed pre- and post-sexing. Statistical analyses were performed using a one-way analysis of variance and Duncan’s test to determine significant differences.

**Results:**

Fresh semen (control) exhibited significantly higher motility (88.45%) compared to T1 (70.94%) and T2 (72.22%), with p < 0.01. Velocity parameters, including curvilinear velocity, were also significantly reduced post-sexing. However, motility levels in sexed samples still exceeded the 40% AI threshold. The 20%-65% gradient demonstrated better performance in maintaining sperm quality compared to the 20%-60% gradient.

**Conclusion:**

Although sexing reduced motility and kinetic parameters, both gradients yielded semen suitable for AI applications. The 20%-65% gradient showed superior results, indicating its potential for optimizing the sexing process. Further research is recommended to refine the technique and improve the viability of sexed sperm.

## INTRODUCTION

Artificial insemination (AI) technology is important for increasing dairy cattle population and production worldwide. AI enables the widespread dissemination of superior genetics, improves the quality and quantity of milk production, and accelerates genetic improvement in livestock [1, 2]. One innovation in this technology is the sexed semen, which increases the probability of female calves being born as replacement stock for females in the dairy industry [3]. Semen sexing allows the separation of spermatozoa based on sex chromosomes, resulting in spermatozoa enriched with X or Y. This technique allows farmers to select female calves for birth, which are more desirable for milk production [4]. In Indonesia, a commonly used sexing method is Percoll density gradient centrifugation (PDGC) [5]. This method uses the density difference between spermatozoa carrying the X and Y chromosomes to effectively separate them. However, the success rate of this technology must be improved. Therefore, optimizing the sexing method is necessary to improve the efficiency and accuracy of spermatozoa separation [6]. One approach is to examine various density gradients used in centrifugation methods [7]. In this context, comparative studies on density gradients are highly relevant. To understand and improve the success of this sexing method, it is essential to observe the kinetic parameters of post-sexing sperm. These parameters include total motility (MOT), progressive MOT (PMOT), velocity curvilinear (VCL), velocity straight line (VSL), mean path velocity, lateral head displacement amplitude of the lateral head (ALH), and beat cross frequency (BCF) [8].

Observation of these kinetic parameters is usually performed using a computer-assisted sperm analysis (CASA), which provides a more objective and detailed analysis than manual methods [9]. Since 1974, the CASA has been available to assess spermatozoa movement and perform kinetic studies objectively. The CASA has analyzed <30 spermatozoa in early versions in 4–8 frames. Today, the CASA can capture images of 20–1000 spermatozoa in more than 30 frames, providing comprehensive motion analysis in <2 min [10]. The CASA can determine the main kinetic parameters of a semen sample: Total and PMOT, VCL, straight-line velocity, mean path velocity average path (VAP), lateral head displacement amplitude, and cross-beat frequency [11]. CASA also provides several ratios, such as mean path straightness (STR; VSL/VAP), curvilinear path linearity (LIN; VSL/VCL), and wobble coefficient (WOB; VAP/VCL) [12]. This information provided by computer-assisted spermatozoa analysis offers the advantage of reducing bias compared with visual evaluation. In addition, CASA allows the definition of spermatozoa subpopulations: Immobile, slow, medium, and fast sperm in each field. Spermatozoa subpopulations are associated with semen quality indicators, such as MOT activation, *in vitro* capacitation, resistance to freezing-induced cell damage, and fertility [13].

The use of CASA is also essential for evaluating the quality of spermatozoa after sexing. Sexing sperm can often cause stress and damage to the sperm, which can affect kinetic parameters and, ultimately, fertilization success [14]. This study aims to evaluate and optimize the spermatozoa sexing method using different PGDC methods and analyze the kinetic parameters of Holstein-Friesian bull spermatozoa to enhance the efficiency and reliability of AI practices. It is expected that the results of this study will increase the effectiveness of AI in the production of Holstein-Friesian dairy cows, especially in terms of increasing the birth of female calves as female replacement stock.

## MATERIALS AND METHODS

### Ethical approval

This study was conducted in full compliance with ethical procedures and standards. All steps were adhered to the Singosari Artificial Insemination Center (SAIC) operational protocols, which are ISO 9001:2015 certified.

### Study period and location

The study was conducted from August to December 2023 at the SAIC Laboratory and the Animal Reproduction Laboratory of the Faculty of Animal Science, Universitas Brawijaya, Malang.

### Study animals

This study used fresh semen from two Holstein-Friesian bulls (ID: Diplomacy and Raja) aged 4 years and 6 years, with body weights between 600 and 870 kg and body condition scores 3.5 and 3, which were kept at the SAIC. Semen collection procedures followed SAIC standards. Fresh semen was collected twice a week, routinely using an artificial vagina (AV; IMV Technologies, France) filled at 40–50°C with 450–500 mL into the AV. The AV was then smeared with a lubricating gel starting from the outside of the hole to ⅓ of the top of the AV and preventing the outside of the AV hole from being touched under sterile conditions. The quality of semen used in this study met the criteria of individual MOT ≥70% and abnormality <20%.

### Study design

This was a laboratory experiment with three treatments with 10 replicates each. Observations were performed on fresh semen after freezing in liquid nitrogen at –196°C and then thawing at 37°C for 30 s. The treatments in this study consisted of three groups: Treatment 0 (T0): Fresh semen before sexing (control); Treatment 1 (T1): Semen sexing with 20%–65% gradient. The diluent used was egg yolk tris aminomethane with a concentration of 80%–35% and Treatment 2 (T2): Semen sexing with 20%–60% gradient. The diluent used in this treatment was egg yolk tris aminomethane with a concentration of 80%–40%. The chemical composition of the Tris egg yolk-based diluent is as follows: Tris aminomethane 1.363 g, citric acid 0.762 g, lactose 1.5 g, fructose 0.5 g, egg yolk 20 mL, raffinose 2.7 g, streptomycin 0.1 g, penicillin 0.1 g, and distilled water 80 g.

### Sexing procedure

The sexing procedure was performed using the PDGC method [15]. Percoll density gradient medium (Merck, St. Louis, MO, USA) was used for cell centrifugation based on density gradients. Percoll consists of colloidal silica particles with a diameter size of 15–30 nm (23% w/w in water) coated with polyvinylpyrrolidone. The diluent used was Tris aminomethane egg yolk. The process started by preparing a Percoll solution and diluent, which were mixed according to the treatment to form a gradient. The number of gradients used was 10. Semen (1 mL) was added slowly over the gradient and centrifuged at 541 × *g* for 5 min. The supernatant (2 mL) containing Y sperm and sediment (2 mL) containing X sperm were each transferred to a tube with diluent and then washed through centrifugation at 286 × *g* for 5 min. The supernatant was referred to as the upper layer because it represented the top layer with a higher concentration of Y-bearing sperm. Conversely, the sediment was referred to as the lower layer, which contained a higher concentration of X-bearing sperm. Then, quality testing and analysis of spermatozoa kinetic parameters were conducted.

### Freezing and thawing

The semen was frozen by placing the semen straw in liquid nitrogen at –196°C until complete freezing. The freezing method used in this study followed a structured stepwise protocol. First, VA1, which is the volume of the extender, was added to the semen at a 1:1 ratio and maintained in a water bath at 37°C. The temperature was then gradually reduced to 5°C. Next, VA2 was added at 12°C–15°C. VA2 was calculated as half of the total volume after subtracting the combined volume of VA1 and the semen sample. After a cooling period of 22 h, VB was added to the samples at a temperature of 3°C–5°C. VB consisted of the combined volumes of VA1 and VA2, along with 13% glycerol, and was calculated as half of the total volume. The semen samples were evaluated for individual MOT to ensure a threshold of ≥55% before proceeding to freezing. The semen was then filled into straws (Filling sealing machine, IMV-France), sealed in a cool tub, and equilibrated under controlled cooling conditions. Pre-freezing was carried out at –140°C for 9 min, and final freezing was achieved by plunging the straws into liquid nitrogen at –196°C, where they were stored for 24 h. The straw was then stored in a container filled with liquid nitrogen. The semen was thawed in water at 37°C for 30 s.

### Kinetic parameters measured using CASA

The process of analyzing semen MOT characteristics was carried out with meticulous care and precision using the CASA IVOS system (CASA Hamilton Thorne IVOS II, France) at SAIC. Sample preparation begun with dripping 3–4 µL of liquid semen on a glass slide (Microscope slide, China) that was warmed at 37°C and then covered with a glass cover (Menzel-Glaser, Germany). The microscope was set to phase contrast at pH 1 and 10 × 10 magnification, and the reflector was coated with a green filter according to the procedure [8]. The diaphragm and light intensity were adjusted to the microscope color standard, and images were taken from five different fields of view. Next, sperm MOT analysis using the CASA IVOS II system was performed by activating the device. The machine was turned on, and the IVOS II application was opened until the optimal temperature of 36°C–38°C. The sample was then loaded into the device by pressing the load button, and the field of view was set using the jog-in and jog-out buttons. The live mode configuration was adjusted to precisely detect the head and tail of the spermatozoa. The focus was then set using the focus setting button, and the image was taken automatically with the auto-capture feature.

### Parameters

The parameters used to measure the quality of spermatozoa in this study were analyzed using CASA, including individual MOT, PMOT, distance straight line (DSL, µm), distance curvilinear (DCL, µm), distance average path (DAP, µm), (VSL, µm/s), (VCL, µm/s), (VAP, µm/s), (STR, %), (LIN, %), (WOB, %), (ALH, µm), and (BCF, Hz).

### Statistical analysis

All data were analyzed using Statistical Package for the Social Sciences (SPSS®) software version 25 (IBM Corp., NY, USA). Prior to analysis, data were subjected to the Shapiro-Wilk test to confirm normal distribution. Normally distributed data were then analyzed using a one-way analysis of variance (ANOVA) to identify significant differences between groups. For post-hoc comparisons, Duncan’s multiple range test was employed to determine specific differences between treatment groups. Results with p ≤ 0.05 were considered statistically significant. Data are presented as mean ± standard deviation for all measured parameters, including motility, velocity, and movement ratios.

## RESULTS

### MOT and velocity parameters (MOT, PMOT, VCL, VAP, and VSL)

Table 1 the percentage of MOT and PMOT were significantly higher (p < 0.05) in the T0 group than in the T1 and T2 groups for the upper and lower layers indicating significant differences between treatments. Duncan’s test further confirmed that T0 had a significantly higher MOT percentage than T1 and T2 (Table 1). This suggests that T1 and T2 treatments, which involved the process of semen separation or sexing based on sex, affected spermatozoa MOT. The decrease in MOT observed in T1 and T2 is consistent with previous studies showing that semen processing techniques can negatively impact or reduce spermatozoa quality.

**Table 1:** Motility and velocity parameters (motility, progressive motility, VCL, VAP, and VSL).

Parameters	Treatment					

	Upper layer ± SD			Lower layer ± SD		
	
	T0	T1	T0	T0	T0	T2
MOT (%)	88.45 ± 10.60^a^	70.94 ± 9.25^b^	72.22 ± 13.55^b^	88.45 ± 10.60^A^	71.70 ± 6.19^B^	69.94 ± 8.43^B^
PMOT (%)	78.16 ± 10.61^a^	40.67 ± 5.72^b^	45.52 ± 11.66^b^	78.16 ± 10.61^A^	45.69 ± 9.03^B^	46.49 ± 5.68^B^
VCL (µm/s)	199.12 ± 22.00^a^	173.37 ± 19.75^bb^	149.17 ± 24.47^c^	199.12 ± 22.00^A^	167.02 ± 13.76^B^	160.82 ± 11.43^B^
VAP (µm/s)	145.48 ± 21.80^a^	92.87 ± 9.22^b^	83.81 ± 9.15^b^	145.48 ± 21.80^A^	90.05 ± 7.67^B^	88.76 ± 7.37^B^
VSL (µm/s)	133.97 ± 20.33^a^	67.86 ± 8.26^b^	64.59 ± 6.38^b^	133.97 ± 20.33^A^	68.29 ± 8.88^B^	68.90 ± 6.46^B^

a,b=similar notation letters on the same line, which means there is no significant difference at the Duncan’s test level and has a value of 5% for the upper layer. A,B=similar letter notation on the same line means no significant difference at the Duncan’s test level and has a value of 5% for the lower layer. MOT=Motility, PMOT=Progressive motility, VSL=Velocity straight line, VCL=Velocity curvilinear, VAP=Velocity average path, SD=Standard deviation

The results of statistical analysis showed that the T0 treatment had significantly higher VCL, VAP, and VSL values than the T1 and T2 treatments for both the upper and lower layers (p < 0.05). In the control group (T0), the VCL value showed a higher spermatozoa velocity speed, with a value of 199.12 ± 22.00 for both layers. In contrast, the T1 and T2 treatment groups showed a significant decrease in VCL, namely, 173.37 ± 19.75 and 149.17 ± 24.47 in the upper layer and 167.02 ± 13.76 and 160.82 ± 11.43 in the lower layer. Duncan’s test confirmed significant differences between T0 and T1, and T2, and between T1 and T2 in the upper layer, indicating that the sexing process can drastically reduce the VCL of spermatozoa. The average velocity of passage (VAP) was also significantly decreased in the treatment groups (Table 1).

### Sperm movement parameters (ALH and BCF)

Table 2 the results (Table 2) showed that there was a significant difference (p < 0.05) between treatments, which was confirmed by the Duncan test, where the T0 group had significantly lower ALH values compared to T1 and T2. Components of the head movement pattern parameters of spermatozoa using CASA include ALH and BCF [12]. ANOVA results showed significant differences (p < 0.05) between treatments, and Duncan’s test showed that the T0 group had significantly higher BCF than the T1 and T2.

**Table 2:** Sperm movement parameters.

Parameters	Treatment					

	Upper layer ± SD			Lower layer ± SD		
	
	T0	T1	T2	T0	T1	T2
ALH (μm)	6.02 ± 0.80^a^	9.34 ± 0.94^b^	8.05 ± 1.52^b^	6.02 ± 0.80^A^	8.88 ± 0.78^B^	8.56 ± 0.82^B^
BCF (Hz)	37.94 ± 2.19^a^	21.87 ± 1.44^b^	23.64 ± 3.22^b^	37.94 ± 2.19^A^	22.80 ± 2.85^B^	22.64 ± 2.94^B^

a,b=similar notation letters on the same line, which means there is no significant difference at the Duncan’s test level and has a value of 5% for the upper layer. A,B=similar letter notation on the same line means no significant difference at the Duncan’s test level and has a value of 5% for the lower layer. ALH=Amplitude of lateral head, SD=Standard deviation, BCF=Beat cross frequency

### Sperm ratio parameters

Table 3 shows a significant difference (p < 0.05) in STR, LIN, and WOB values between the control group (T0) and the treatment group (T1 and T2) in both the upper and lower layers. Duncan’s test further confirmed that the STR, LIN, and WOB values in the control group (T0) were significantly higher than those in the treatment groups (T1 and T2).

**Table 3:** Sperm ratio parameters.

Parameters	Treatment					

	Upper layer ± SD			Lower layer ± SD		
	
	T0	T1	T2	T0	T1	T2
STR (%)	92.09 ± 1.92^a^	73.12 ± 5.19^b^	77.34 ± 5.88^b^	92.09 ± 1.92^A^	76.06 ± 5.53^B^	77.66 ± 4.03^B^
LIN (%)	67.20 ± 5.38^a^	39.33 ± 4.04^b^	55.21 ± 7.65^b^	67.20 ± 5.38^A^	41.15 ± 4.76^B^	42.89 ± 3.29^B^
WOB (%)	72.94 ± 5.14^a^	53.68 ± 2.09^b^	56.83 ± 5.36^b^	72.94 ± 5.14^A^	53.98 ± 3.02^B^	55.18 ± 2.11^B^

a, b=similar notation letters on the same line, which means there is no significant difference at the Duncan’s test level and has a value of 5% for the upper layer. A, B=similar letter notation on the same line means no significant difference at the Duncan’s test level and has a value of 5% for the lower layer. SD=Standard deviation, STR=Straightness, LIN=Linearity, WOB=Wobble

### Distance parameters (DCL, DAP, and DSL)

**Figure 1:**
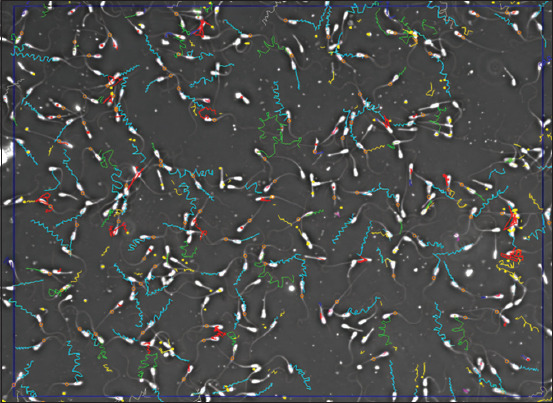
Trajectory pattern of sexed spermatozoa after thawing; green: Motile, light blue: Progressive, pink: Slow, red: Static, dark blue: Border crosser, yellow: Late track, gray: Late entry.

Table 4 shows significant differences (p < 0.05) in DCL, DAP, and DSL parameters among the treatment groups (T0, T1, and T2) for both the top and bottom layers. Duncan’s test further revealed that the values in the control group (T0) were significantly higher than those in the treatment groups (T1 and T2) for all three distance parameters. DCL, DAP, and DSL parameters are essential indicators of sperm MOT ability that reflect the efficiency and effectiveness of spermatozoa movement (Figure 1).

**Table 4:** Distance parameters.

Parameters	Treatment					

	Upper layer ± SD			Lower layer ± SD		
	
	T0	T1	T2	T0	T1	T2
DCL (μm)	74.66 ± 8.33^a^	64.38 ± 6.84^b^	58.32 ± 7.80^b^	74.66 ± 8.33^A^	67.55 ± 5.24^AB^	59.94 ± 14.25^B^
DAP (μm)	54.65 ± 8.01^a^	33.96 ± 4.33^b^	32.43 ± 2.98^b^	54.65 ± 8.01^A^	35.91 ± 2.94^B^	34.86 ± 3.60^B^
DSL (μm)	49.98 ± 6.96^a^	24.61 ± 3.79^b^	24.91 ± 2.83^b^	49.98 ± 6.96^A^	27.15 ± 3.62^B^	26.98 ± 3.66^B^

a, b=similar notation letters on the same line, which means there is no significant difference at the Duncan’s test level and has a value of 5% for the upper layer. A, B=similar letter notation on the same line means no significant difference at the Duncan’s test level and has a value of 5% for the lower layer. DSL=Distance straight line, DCL=Distance curvilinear, DAP=Distance average path, SD=Standard deviation

## DISCUSSION

The decrease in MOT observed in T1 and T2 is consistent with previous studies showing that semen processing techniques can negatively impact or reduce spermatozoa quality. Sharafi *et al*. [16] reported that sex separation of spermatozoa involves stress factors, such as centrifugation and staining processes, which can lead to decreased MOT and cell viability in spermatozoa. In addition, the reduction in MOT may be attributed to structural changes in spermatozoa flagella caused by centrifugation [17]. A progressive decrease in MOT was also observed in the T1 and T2 groups. Cunha *et al*. [18] reported that spermatozoa processed through a density gradient exhibit a linear decrease in swimming ability, which is essential for passage through the female reproductive tract. This decrease could be due to damage to mitochondria, which are the primary source of adenosine triphosphate (ATP) for spermatozoa movement [19]. Separating sperm by Percoll density gradient involves several steps that can cause stress to the sperm cells [7]. These steps include high-speed centrifugation, osmotic changes, and contact with Percoll media, which can affect the integrity of the spermatozoa membrane and other intracellular components [20]. According to Morrell and Rodriguez-Martinez [21], centrifugation can cause mechanical damage to the plasma membrane of spermatozoa, disrupt mitochondrial function, and increase the production of reactive oxygen species (ROS). Excess ROS can lead to membrane lipid peroxidation, disrupt spermatozoa MOT, and reduce viability [22].

The VCL measures the total distance traveled by the spermatozoa, considering the trajectory taken during movement. A high VCL indicates that spermatozoa have sufficient kinetic energy to rush through the female reproductive tract and reach the oocyte. However, in this study, T1 and T2 treatments resulted in a significant decrease in VCL compared with the control group (T0). The decrease in VCL is related to damage to the plasma membrane structure of spermatozoa during the centrifugation process [23]. According to Crespo-Félez *et al*. [24], centrifugation can induce mechanical stress, damaging the spermatozoa membrane and mitochondria, which are energy-producing centers for spermatozoa movement. In addition, differences in sperm density during the density gradient process can cause specific fractions of sperm to suffer more significant damage from the applied centrifugal force [25]. Damage to mitochondria inhibits the production of ATP, an energy molecule required for flagella movement, which directly decreases VCL. Ferramosca and Zara [26] confirmed that impaired mitochondrial function can lower VCL by reducing the efficiency of ATP production, which is essential for spermatozoa MOT. Physical and chemical stresses during sexing disrupt cell membrane stability and alter spermatozoa movement patterns. Garner and Seidel [27] explain that staining and centrifugation can trigger increased oxidative stress, leading to membrane lipid peroxidation and reduced spermatozoa MOT. Oxidative stress increases the production of free radicals that damage lipids, proteins, and DNA in spermatozoa cells, disrupting efficient movement patterns [28]. The centrifugation process can disrupt axonemal microtubules, inhibiting the propulsive movement of spermatozoa and decreasing VSL [29]. In addition, changes in the ionic environment during separation can affect the stability of the axoneme structure, resulting in a decrease in the efficiency of linear movement in spermatozoa [30].

ALH measures the amplitude of the spermatozoa head’s lateral movement and is a critical parameter in spermatozoa MOT analysis [31]. The baseline ALH measurements were taken from T0, which consisted of fresh, unsexed semen. This control group represents the natural MOT of sperm. In contrast, groups T1 and T2 underwent sperm sexing, potentially introducing physical and chemical stresses to the sperm cells through methods such as centrifugation, which involves exposure to physical forces. The observed changes in ALH values between the control (T0) and treatment groups (T1 and T2) indicate a significant effect of the sexing process on sperm MOT. Higher ALH values in T1 and T2 suggest a state of hyperactivation, possibly induced by the handling and processing involved in sperm. Conversely, a decrease in ALH levels indicates potential sperm damage, reducing the efficacy of lateral head movements. High ALH is often associated with hyperactive movement of the spermatozoa, which is an essential phenomenon in the capacitation process, where the spermatozoa undergo a series of biochemical changes that enhance their ability to fertilize eggs [32]. The hyperactive movement indicated by increased ALH levels is essential for fertilization, especially for the penetration of the oocyte zona pellucid. According to Nabilla *et al*. [33], hyperactive movement, characterized by higher ALH values, allows spermatozoa to interact more effectively with the oocyte protective membrane, which is a critical fertilization step. Belala *et al*. [34] showed that the ideal ALH value for improving fertility potential is between 2.5 and 6.5 µm. The value indicates a balance between efficient movement and hyperactivation required for oocyte penetration. In this study, an increase in ALH in the T1 and T2 groups indicates physiological changes that could support improved fertilization ability; an excessive increase could indicate cellular stress that could reduce quality. According to Waberski *et al*. [35], BCF and ALH parameters evaluated using CASA provide deep insight into spermatozoa’s movement patterns and MOT dynamics. High BCF values are associated with efficient use of energy by spermatozoa [36], as spermatozoa with higher BCF tend to have more optimized energy metabolism, allowing them to reach the oocyte more effectively [37].

STR and LIN percentages are used to identify sperm movement patterns, including the identification of hyperactive sperm that move quickly and firmly but are not progressive and linear [38]. In this study, the higher STR and LIN values in the control group (T0) indicated that sperm moved more linearly and directionally. The LIN of sperm movement is an essential indicator of the sperm ability to reach the oocyte with maximum efficiency [9]. Higher LIN, as observed in the T0 group, indicates better movement direction and optimal swimming STR, increasing the chance of fertilization. Conversely, a decrease in STR and LIN values in the treatment groups (T1 and T2) suggests that the sexing process may have disrupted the spermatozoa movement’s movement pattern and efficiency. Spermatozoa are considered to move linearly if they exhibit STR >50% and LIN >35% [33, 39]. In this study, although the T1 and T2 groups decreased, the STR value remained above 50% and the LIN value was >35%, indicating that spermatozoa can still move linearly. Křížková *et al*. [31] reported that spermatozoa are progressive when the STR percentage is >75%. In this study, the control group (T0) and treatment (T1 and T2) groups exhibited an average STR of >75%, indicating a progressive and directed movement toward the oocyte. However, T0 exhibited a significantly higher STR than T1 and T2. In contrast, the treatment groups (T1 and T2) showed a decrease in progressivity. The balance between stability and flexibility of spermatozoa movement indicated by high WOB values is the key to successful fertilization-WOB measures oscillation/back-and-forth movement on the actual trajectory [40].

DCL measures the total path traveled by sperm during movement, providing an idea of the extent to which sperm can move in complex and winding patterns. High DCL values indicate that sperm move extensively [41]. DAP measures the distance sperm travel along an average path [42]. The DSL reflects the shortest distance between the spermatozoa movement’s start and end points [43]. A decrease in DSL indicates that sperm has difficulty in moving to the Zona pellucid [44]. The zona pellucid is the protective layer around the oocyte that sperm must penetrate for successful fertilization. The sperms’ ability to move in a straight line with sufficient speed is essential for penetrating this layer [45]. The significant differences in DCL, DAP, and DSL values between the control and treatment groups indicate that the spermatozoa sexing process lowers spermatozoa MOT. According to Liu and Li [46], the separation process, which often involves centrifugation and freezing processes, can cause mechanical stress to sperm, affecting their structural and functional integrity. These physical stresses can damage the sperm cell membrane and alter the dynamics of flagellum movement, reducing the efficiency of sperm movement and navigational ability [47]. Damage to the flagellum structure can inhibit the sperm ability to produce progressive movements to reach the oocyte [5]. According to Gilmore *et al*. [48], a high DCL value indicates that spermatozoa move faster and is correlated with VCL. A high DCL value follows a high VCL value. This is because the velocity value is positively correlated with the distance value. The DAP value correlates with the VAP value, whereas a high DCL value decreases the DAP value [49]. Spermatozoa with optimal DCL, DAP, and DSL values are more likely to reach and penetrate the oocyte, increasing the chance of successful fertilization and live birth [50].

## CONCLUSION

This study evaluated the effectiveness of PDGC for sperm sexing and its impact on the kinetic parameters of Holstein-Friesian bull spermatozoa. The findings revealed that while the sexing process resulted in a significant reduction in motility and velocity parameters, semen processed with both 20%–65% and 20%–60% density gradients maintained motility levels above the 40% threshold required for artificial insemination. The 20%–65% gradient demonstrated better preservation of sperm quality compared to the 20%–60% gradient, with higher values for motility (70.94% vs. 72.22%) and curvilinear velocity (173.37 µm/s vs. 149.17 µm/s).

The study employed a robust and objective approach using CASA, which minimizes observer bias and provides detailed insights into sperm movement and functionality. Additionally, the use of standardized methods and replicates ensures reproducibility and reliability of results. However, a limited sample size of two Holstein-Friesian bulls may not fully capture the variability in sperm characteristics across a larger population. Furthermore, the study focused on short-term kinetic parameters and did not evaluate the long-term fertilization success of sexed sperm.

Future research should expand the sample size and include diverse breeds to generalize the findings. Investigations into the biochemical and structural changes in spermatozoa induced by the sexing process could offer insights into improving sperm viability and reducing oxidative stress. Long-term studies assessing pregnancy rates, calving outcomes, and the economic feasibility of the PDGC method are recommended to optimize its practical application in dairy farming. This study highlights the potential of the 20%-65% PDGC gradient in improving sexed semen quality while identifying areas for further research to enhance the efficiency and applicability of the technique.

## AUTHORS’ CONTRIBUTIONS

PU, CNAS, and HAS: Conceptualized and designed the study, collected and analyzed data, and drafted and revised the manuscript. APAY and AA: Conceptualized and designed the study and reviewed the manuscript. TAS, NI, and TS: Supervised and designed the study and reviewed the manuscript. All authors have read and approved the final manuscript.
